# Stevens-Johnson Syndrome From Combined Allopurinol and Angiotensin-Converting Enzyme Inhibitors: A Narrative Review

**DOI:** 10.7759/cureus.51899

**Published:** 2024-01-08

**Authors:** Isabella M Fabian, Kirsten Maddox, Cameron Robicheaux, Rahib K Islam, Ahmed Anwar, Bradley Dorius, Christopher L Robinson, Adam M Kaye, Giustino Varrassi, Shahab Ahmadzadeh, Sahar Shekoohi, Alan D Kaye

**Affiliations:** 1 School of Medicine, Louisiana State University Health Sciences Center, Shreveport, USA; 2 School of Medicine, Louisiana State University Health Sciences Center (LSUHSC) New Orleans, New Orleans, USA; 3 Department of Psychology, Quinnipiac University, Hamden, USA; 4 Department of Anesthesiology, Louisiana State University Health Sciences Center, Shreveport, USA; 5 Department of Anesthesiology, Critical Care, and Pain Medicine, Beth Israel Deaconess Medical Center, Harvard Medical School, Boston, USA; 6 Department of Pharmacy Practice, Thomas J. Long School of Pharmacy and Health Sciences University of the Pacific, Stockton, USA; 7 Department of Pain Medicine, Paolo Procacci Foundation, Rome, ITA

**Keywords:** angiotensin converting enzyme inhibitors, drug reaction, ace inhibitors, allopurinol, stevens-johnson syndrome

## Abstract

Stevens-Johnson syndrome (SJS) is a severe and potentially debilitating skin reaction frequently related to medication use. Allopurinol and angiotensin-converting enzyme (ACE) inhibitors are commonly prescribed medications for prevalent health conditions worldwide, and their interaction associated with SJS warrants further investigation. A comprehensive literature search was performed to investigate cases as studies related to SJS occurring in patients with concomitant use of allopurinol and ACE inhibitors. We identified case reports and studies detailing hypersensitivity reactions, including SJS, attributed to a combination of allopurinol and ACE inhibitors. Despite the drug-drug interactions or lack thereof seen in patient populations, there is no definitive evidence of a pharmacokinetic interaction between allopurinol and ACE inhibitors. We were only able to find one case report specifically detailing SJS in a patient on combined ACE inhibitors and allopurinol. While the exact mechanism of the interaction is unclear, those reported cases of severe hypersensitivity reactions suggest a previous history of impaired renal function as a predisposing factor in the development of SJS. The potential risk of SJS with coadministration of ACE inhibitors and allopurinol is a drug-drug interaction that physicians should be aware of. This topic requires additional attention to determine if this drug combination should be avoided entirely in certain patients.

## Introduction and background

Stevens-Johnson syndrome (SJS) is a rare and serious skin reaction characterized by detachment of epidermal and mucosal surfaces. This condition is typically a result of a response to medications and exists on a spectrum with toxic epidermal necrolysis (TEN). A reaction is classified as SJS if 10% or less of the body surface area is affected, or TEN if 30% or more of the body surface area is affected. Patients with 10% to 30% of their total body surface affected fall within the overlapping zone between SJS and TEN. A few of the most common medication classes known to cause SJS are antibiotics and antiepileptics [1-4]. An online search of the literature demonstrates many case reports of combined allopurinol and ACE inhibitors triggering severe hypersensitivity reactions, including SJS, and advises caution in using said medications concomitantly [5-10]. Additionally, allopurinol and ACE inhibitors are two frequently prescribed medications for common health conditions. It is important to know how they interact. In the present investigation, therefore, we examine the literature to determine if there is a significant association between combining allopurinol and ACE inhibitors and an increased incidence of SJS.

## Review

Stevens-Johnson syndrome

SJS is both an adverse and rare skin condition. In this condition, sheet-like skin and mucosal loss are accompanied by systemic symptoms. Medications are a frequent cause of this syndrome. SJS is classified by the extent of the detached skin surface area. SJS exists on a spectrum defined by the percentage of body surface area affected. SJS is diagnosed when the affected body surface area is 10% or less. When the affected area ranges from 10% to 30%, it represents an overlap between SJS and TEN. TEN is diagnosed when more than 30% of the body's surface area is affected [3,11-15]. SJS is characterized clinically by its cutaneous involvement with a prodrome of symptoms, including fever, malaise, sore throat, and a cough. Subsequent mucosal involvement is nearly universal. Flaccid blisters with a positive Nikolsky sign and sheets of denuded epidermis are present as well. Oral and ocular involvement is also present in many cases [16-19].

Various drug classes contribute to SJS/TEN development. In an 82-patient study, with half experiencing drug-induced SJS, the common drugs, in descending order, were as follows: anticonvulsants (14.6%), antibiotics (11%), non-steroidal anti-inflammatory drugs (NSAIDs; 6.1%), herbal remedies (6.1%), allopurinol (2.4%), and antipsychotics (1.2%). Etiologies included proton pump inhibitors, famciclovir, L-cysteine, and hair dye. Carbamazepine (8.5%) was the primary causative drug. Patients ceased the suspected drug upon SJS/TEN diagnosis in this study [20]. Other studies found the culprit drugs that were at high risk for triggering SJS were phenytoin, lamotrigine, and allopurinol [21,22]. An increased risk for developing SJS is associated with factors such as age, autoimmune disease, previous drug allergies, epilepsy, or a history of cerebrovascular accident [23-25].

There are multiple postulations about the pathogenesis of SJS. The tissue damage in SJS is a result of mass keratinocyte cell death achieved through apoptosis. Stimuli that induce apoptosis include cellular stress, DNA damage, and intracellular cytokines. It is hypothesized that SJS involves drug-specific CD8+ cytotoxic lymphocytes, as well as the Fas-Fas ligand (FasL) pathway of apoptosis. It also involves granule-mediated exocytosis and tumor necrosis factor-alpha (TNF-alpha), a death receptor pathway. The main cause of keratinocyte apoptosis is granulysin, which is found in cytotoxic granules. Fas-FasL is expressed on the activated cytotoxic T cells and can destroy keratinocytes by the production of intracellular caspases [26]. Another proposed mechanism is when cytotoxic T-cells exocytose perforin and granzyme B. This creates a channel in the target cell and activates caspases [27-30]. Another theory of the SJS pathophysiology is slow acetylation, a drug metabolic disorder that results in increased production of toxic reactive metabolites that can trigger a secondary immune response [30-32]. Genetic susceptibility can increase the risk of SJS in certain populations. A robust correlation exists between HLA-B75 and HLA-B15:21 and SJS associated with the use of carbamazepine [33,34]. A high percentage of patients with allopurinol-induced SJS have been found to carry the HLA-B 5801 allele [35,36].

The most accepted intervention for SJS is supportive care. Adjuvant therapies are used in the most severe cases of SJS. The use of systemic corticosteroids as the sole therapy is not supported. The current standard of care for most centers is intravenous immunoglobulin (IVIG). Cyclosporine and TNF inhibitor studies are promising for treating SJS. Both IVIG and cyclosporine have been associated with better healing as well as better survival because of their ability to inhibit apoptosis [11,27,37]. Survivors of SJS may develop delayed sequelae, which can be associated with significant morbidity. The respiratory tract and gastrointestinal tract can be affected. Long-term problems can also include impaired taste, difficulty urinating, and genital abnormalities. Alterations in skin coloration, dryness of the skin, and mucous membranes, along with hair loss, are also observed [38,39,40]. Multiple recurrences of SJS are rare and occurred in 1.4% of cases in a 10-year population-based cohort study [41]. The average mortality of SJS has been reported anywhere from 1.9% to 34%. Predictors of mortality include increasing age, number of chronic conditions, infection, hematological malignancy, and renal failure [42-45].

Allopurinol

Allopurinol is a xanthine oxidase inhibitor used primarily to lower urate levels. It is metabolized in the liver and becomes its pharmacologically active form, oxypurinol. Both allopurinol and oxypurinol inhibit an enzyme called xanthine oxidase, which is involved in the breakdown of purines to uric acid. Allopurinol has a short half-life of one to two hours, while oxypurinol has a longer half-life of around 15 hours. Both drugs are excreted from the body through the kidneys [46-48]. Allopurinol is approved by the Food and Drug Administration (FDA) for the use, management, and treatment of a plethora of conditions involving gout, tumor lysis syndrome, and recurrent calcium nephrolithiasis in patients with hyperuricosuria [49-50]. Other indications for allopurinol involve hyperuricemia involved with Lesch-Nyhan syndrome and the prevention of recurrent uric acid nephrolithiasis [51,52]. Furthermore, guidelines delineated by the American College of Rheumatology recommend the usage of allopurinol in patients with conditions that involve frequent attacks of acute gouty arthritis, defined as equal to or more than two attacks per year, chronic kidney disease stage 2 or worse, presence of tophi or tophus on clinical exam or imaging, and history of nephrolithiasis [53]. Various side effects come with the usage of allopurinol. In a case study, two patients displayed symptoms of fever, dermatitis, renal insufficiency, and eosinophilia, following the initiation of allopurinol therapy. The clinical, laboratory, and histologic findings were consistent with a severe hypersensitivity reaction. Both patients necessitated long-term corticosteroid treatment to manage their symptoms. These results indicate that hypersensitivity to allopurinol can lead to a severe, prolonged, or potentially life-threatening condition [54].

SJS is a side effect that occurs with the ingestion of allopurinol. SJS is labeled as a severe cutaneous adverse reaction (SCAR), and SCARs are defined as a form of a hypersensitivity reaction that is both severe, unpredictable, and induced by a drug [55,56]. In a multinational case-control study spanning from 1997 to 2001 in Europe, the potential of medications to elicit SCARs was meticulously examined. Cases were proactively identified via an extensive hospital network covering over 100 million patients. Controls were selected to match cases based on age, gender, and date of interview, with three hospitalized patients per case. After verification by an expert committee that was blinded to exposures, 379 SCAR cases and 1,505 controls were included for analysis. The results confirmed that there was a robust correlation between allopurinol, one of the drugs tested, and the occurrence of SCARs, albeit with some fluctuations in the relative prevalence of exposed cases. Furthermore, several widely tested drugs did not show any risk for the occurrence of SJS [57].

Furthermore, other studies revealed that there are patient-specific factors that make it more likely to get SJS when taking allopurinol. A retrospective nationwide population study that used data from the Taiwan National Health Insurance Research Database occurred from January 1, 2005, to December 31, 2011. From this study, it was revealed that having a female gender, an age of 60 years or more, an initial allopurinol dose of more than 100 mg/day, and the presence of renal or cardiovascular disease states were identified as risk factors for allopurinol hypersensitivity. Additionally, the use of allopurinol for treating asymptomatic hyperuricemia conferred a higher risk of developing allopurinol hypersensitivity [58].

ACE inhibitors

ACE inhibitors are medications mainly used to treat and manage hypertension [59-61]. Captopril is an ACE inhibitor widely used to treat patients with hypertension. The adverse effects noted during the administration of captopril and other ACE inhibitors can be categorized according to their clinical significance. These include significant hematological toxicity, such as neutropenia; renal toxicities leading to either renal functional impairment or glomerular dysfunction resulting in proteinuria; minor side effects like rash and dysgeusia; and those linked to excessive drug action, such as hypotension and hyperkalemia [62,63]. Patients with renal impairment, collagen vascular disease, or those undergoing immunosuppressive therapy are at higher risk for certain side effects such as neutropenia. However, in uncomplicated patients, the risk is small and similar to standard treatments. Minor side effects are infrequent, temporary, and reversible, and those related to ACE inhibition can be prevented except in cases of underlying and clinically significant renovascular disease [64-68]. There is an association between SJS and the use of ACE inhibitors, although not a strong association. In a study done by Mockenhaupt et al., less than 100 case reports were recorded that involved the use of ACE inhibitors [58].

Allopurinol and ACE inhibitors, interactions, and SJS

There have been several reported instances of adverse reactions following the coadministration of allopurinol and ACE inhibitors with manifestations ranging in severity. Some effects are mild and limited to fever, arthralgia, myalgias, and exfoliative dermatitis [69]. However, cases exist of more severe reactions. Such interactions can manifest as SJS, with one fatality reported three to five weeks after administration of allopurinol and an ACE inhibitor [7].

Among the 10 drugs in the class of ACE inhibitors, many of the reported interactions have been seen with the administration of captopril [69,70]. One study analyzing drug-drug interactions in an Indonesian hospital showed a prevalence of three adverse reactions over five months in those taking captopril and allopurinol. While two of the three did not suffer from hypersensitivity reactions, one did experience kidney failure [70]. Further, a study involving elderly patients with arterial hypertension reported eight cases of severe drug-drug interactions between unspecified ACE inhibitors and allopurinol [71]. While most reported cases involve captopril, there does exist an isolated case report involving enalapril in a man who experienced a severe hypersensitivity reaction as a result of allopurinol administration following a long-standing use of enalapril. Within 20 minutes of 100 mg allopurinol administration, he manifested with anaphylactic symptoms of generalized pruritus, urticaria, severe chest pain, severe nausea, peripheral cyanosis, hypotension, sinus tachycardia, mild bronchospasm, and elevated cardiac enzymes, suggesting a myocardial infarction. Once stabilized, the patient continued his normal enalapril regimen with no issues, suggesting the administration of allopurinol as the primary culprit leading to the adverse reaction [8].

While reported adverse events exist, there is evidence that could suggest the opposite. A study conducted on 12 healthy individuals showed no change in the bioavailability of captopril following administration of allopurinol [72]. Further, the literature suggests that ACE inhibitors, when used in conjunction with allopurinol, can benefit patient outcomes. They can act synergistically in treating conditions like fructose-induced metabolic syndrome, leading to a significant reduction in blood pressure, less accumulation of abdominal fat, and improvements in dyslipidemia and even total prevention of insulin resistance. This suggests that the coadministration of ACE inhibitors and allopurinol could be promising in diabetes prevention and cardiovascular conditions. Despite the drug-drug interactions or lack thereof seen in patient populations, there has been no evidence produced that identifies a pharmacokinetic interaction between allopurinol and ACE inhibitors. While the exact mechanism of the interaction is unclear, those reported cases of severe hypersensitivity reactions suggest a previous history of impaired renal function to be a predisposing factor. With that, it has been speculated that the adverse reactions following allopurinol administration with concomitant ACE inhibitor use are due to a stand-alone hypersensitivity to allopurinol itself. Allopurinol certainly has been known to cause severe side effects, including SJS, in certain patient populations. Interestingly, those reactions isolated to allopurinol use can be seen in those with a prior history of renal failure [73]. Further, one study notes that allopurinol alone is the most common cause of SJS in Europe and Israel [22]. Thus, it may be possible that taking an ACE inhibitor could be the result of an unrelated hypersensitivity reaction to allopurinol, and perhaps the administration of an ACE inhibitor may enhance the potential for allergic/hypersensitivity reactions to allopurinol [71].

Both allopurinol and ACE inhibitors have individually been known to cause SJS. A 2016 review utilizing the Japanese adverse event database reported that SJS/TEN comprised 544/2,642 (20.59%) adverse events seen following the administration of allopurinol. A multinational case-control study identified allopurinol as the most common cause of SJS and TEN in Europe and Israel [22]. Of note, the onset of SJS following allopurinol administration did not predominantly affect any particular age group, which cannot be said of every SJS-causing drug [74].

Mockenhaupt et al. identified adverse reactions seen following the administration of ACE inhibitors. In this study, ACE inhibitors encompassed imidapril hydrochloride, enalapril maleate, temocapril hydrochloride, lisinopril hydrate, perindopril erbumine, and alacepril. From these data, it appears that SJS/TEN is far less prevalent following administration of ACE inhibitors than allopurinol, as only 44/379 (11.6%) patients in the study taking ACE inhibitors developed SJS/TEN [58].

Interestingly, a study reports that the onset of SJS/TEN following allopurinol administration occurs within a median of 21 days, whereas the median for ACE inhibitors is 20 days. With a similar onset of SJS for each of these drugs, it may be difficult to determine if an adverse reaction is due to their administration or their coadministration [74].

Discussion

Through our review of the literature, we have found many reports of hypersensitivity reactions but only one report of a patient experiencing SJS while being treated with an ACE inhibitor and allopurinol simultaneously. The interaction between the drugs and the reason for this adverse drug reaction remains unclear. SJS is a severe hypersensitivity reaction that may require extensive treatment and close monitoring, and interactions that could induce such a side effect should be avoided if possible. Allopurinol, a xanthine oxidase inhibitor commonly used to treat gout and other disorders, is a relatively common cause of SJS. In contrast, ACE inhibitors, a mainstay treatment for heart failure and hypertension, are not frequently associated with SJS. These drugs should be administered with caution, and the potentially life-threatening implications should be considered when prescribing allopurinol and ACE inhibitors together. Because recent literature indicates a beneficial synergism with the coadministration of ACE inhibitors and allopurinol in the prevention of diabetes and treatment of cardiovascular conditions, there may be instances that warrant the necessary use of these two medications despite the potential adverse effect of SJS. This provides further reasoning for dedicating more focus to determining the interaction between these drugs and the significance of past medical history that predisposes certain patients to SJS.

## Conclusions

Though allopurinol has been known to cause SJS in some patients, the potential added risk of ACE inhibitor coadministration is an interaction that physicians should be aware of to treat patients safely. This topic requires additional awareness and attention to determine if this drug combination should be avoided entirely, avoided in certain patients, or even intentionally added in the case of diabetes prevention and CV disease treatment. These medications are used frequently, and additional information regarding their involvement with SJS could largely adjust the indications for their use.
